# Juvenile stress enhances anxiety and alters corticosteroid receptor expression in adulthood

**DOI:** 10.1002/brb3.182

**Published:** 2013-10-30

**Authors:** Nichola M Brydges, Rowen Jin, Jonathan Seckl, Megan C Holmes, Amanda J Drake, Jeremy Hall

**Affiliations:** 1 Centre for Cardiovascular Science QMRI, University of EdinburghEdinburgh, U.K; 2 Division of Psychiatry Centre for Clinical Brain Science, University of EdinburghEdinburgh, U.K; 3 Institute of Psychological Medicine and Clinical Neurosciences, Cardiff University School of MedicineCardiff, U.K

**Keywords:** Anxiety, glucocorticoid receptor, juvenile stress, mineralocorticoid receptor, sex differences

## Abstract

**Background:**

Exposure to stress in early life is correlated with the development of anxiety disorders in adulthood. The underlying mechanisms are not fully understood, but an imbalance in corticosteroid receptor (CR) expression in the limbic system, particularly the hippocampus, has been implicated in the etiology of anxiety disorders. However, little is known about how prepubertal stress in the so called “juvenile” period might alter the expression of these receptors.

**Aims:**

Therefore, the aim of this study was to investigate how stress experienced in the juvenile phase of life altered hippocampal expression of CRs and anxiety behaviors in adulthood.

**Materials and methods:**

We used a rodent model to assess the effects of juvenile stress on hippocampal CR expression, and performance in three behavioral tests of anxiety in adulthood.

**Results:**

Juvenile stress (JS) increased anxiety-like behavior on the elevated plus maze, increased mineralocorticoid receptor (MR) expression, and decreased the ratio of glucocorticoid receptor (GR) to MR expression in the hippocampus of adult animals. Females demonstrated lower levels of anxiety-type behavior and increased activity in three behavioral tests, and had greater expression of GR and GR:MR ratio than males, regardless of treatment.

**Discussion and conclusion:**

These results demonstrate that JS can alter the expression and balance of CRs, providing a potential mechanism for the corresponding increase in anxiety behavior observed in adulthood. Further evidence for the role of CR expression in anxiety is provided by sex differences in anxiety behavior and corresponding alterations in CR expression.

## Introduction

In humans, the risk of developing neuropsychiatric disorders such as posttraumatic stress disorder (PTSD), depression, and anxiety in adulthood is increased when stress is experienced earlier in life (Anda et al. 2006; Heim et al. 2008; Bale et al. 2010; Meewisse et al. 2011; Pechtel and Pizzagalli 2011). Major mediators of the effects of early life stress are thought to be corticosteroid hormones and their receptors in the brain (glucocorticoid receptors [GR] and mineralocorticoid receptors [MR]). During a stress response, glucocorticoids (mainly corticosterone in rodents and cortisol in humans) are released as a consequence of activation of the hypothalamic–pituitary–adrenal (HPA) axis. As these stress hormones can pass through the blood–brain barrier, the HPA axis is one of the major pathways through which stress can alter brain development. Indeed, previous work suggests that prenatal stress can program the HPA axis, and may be related to adult pathophysiology (Meaney et al. 2007; Seckl and Holmes 2007).

In the central nervous system, GR and MR receptor densities are highest in the hippocampus (Herman 1993). The hippocampus is an important regulator of behavioral measures of anxiety (Mirescu et al. 2004), and clinical and basic research has identified alterations in the hippocampus in mood disorders (Mayberg 2009; Arnone et al. 2012). Early life stress can structurally and functionally alter the hippocampus (Fenoglio et al. 2006; Tottenham and Sheridan 2010), and stress in the prenatal and neonatal phases alters MR and GR expression in adult animals (rats, primates, and birds). However, the direction of change varies with the exact paradigm used and between sexes, and many effects are GR or MR specific (Welberg et al. 2001; Kapoor et al. 2006; Patel et al. 2008; Lupien et al. 2009; Belay et al. 2011; Wynne et al. 2011; Banerjee et al. 2012; van Hasselt et al. 2012).

Although there is a wealth of information on the adulthood consequences of perinatal stress, comparatively little is known about the effects of juvenile (prepubertal or childhood) stress. The juvenile brain experiences dramatic changes in structure and function as it matures (Romeo and McEwen 2006), and epidemiological studies have linked juvenile stress (JS) with the development of depression, anxiety, and PTSD, as well as suicide attempts later in life (Morgan et al. 2003; Kausch et al. 2006; Weich et al. 2009). In animal models, JS increases anxiety behavior, alters fear conditioning, learning, and memory (Avital and Richter-Levin 2005; Toledo-Rodriguez and Sandi 2007; Tsoory et al. 2007; Jacobson-Pick and Richter-Levin 2010; Brydges et al. 2012, 2013), remodels corticolimbic architecture (Eiland et al. 2012), and alters neural gene expression in adulthood (Jacobson-Pick et al. 2008; Tsoory et al. 2010). Effects on behavior are observed when animals experience stress in adulthood, but they are significantly enhanced when stress is given in the juvenile phase (Avital and Richter-Levin 2005; Tsoory and Richter-Levin 2006), demonstrating phase specific changes.

To date, the effects of JS on the expression of corticosteroid receptor (CR) expression in the adult brain have not been investigated. Therefore, the aims of this study were to investigate the effects of JS on anxiety behavior, and analyze corresponding changes in hippocampal CR expression in adulthood. We hypothesized that JS would increase anxiety behavior in adulthood, and alter the expression of MR and GR. To date, most rodent studies have used rat models to investigate the effects of JS (see Peleg-Raibstein and Feldon 2011, for an exception), but given the large number and availability of transgenic mouse models for the study of genetic components of psychiatric disorders, we aimed to expand this research through use of a mouse model.

## Material and Methods

### Ethics statement

All procedures were carried out in strict accordance with and permission of the local ethics committee, and under the aegis of the UK Home Office Animals (Scientific Procedures) Act, 1986.

### Animals

C57BL/6 mice were bred from eight stressed and seven control adult pairs (Harlan, Oxfordshire, U.K.) at the University of Edinburgh. After weaning (postnatal day [PND] 21), 22 female and 23 male offspring were housed in standard, same-sex, same-litter cages lined with wood shavings (Lillico, Hookwood, Surrey, U.K.), on a 12:12 h light/dark cycle with food (RM1 diet, Special Diet Services, Witham, U.K.) and water provided ad libitum. Humidity and temperature were maintained at 60% and between 19°C and 21°C, respectively. Eight litters were randomly assigned to JS, the other seven served as controls. Age and sex ratios were evenly distributed between the groups.

### JS protocol

The JS protocol follows that used in Brydges et al. (2012, 2013). Eight litters were exposed to variable short-term stress on PND 25, 26, and 27. On PND 25, animals were given a forced swim in a swim tank (15 cm high, 11 cm diameter, 1 L capacity filled with 500 mL water, water temperature 25 ± 1°C) for 10 min. On PND 26, animals received restraint stress; they were placed into plastic restraint tubes (26 mm diameter) for three sessions of 30 min, separated by 30 min breaks. On PND 27, they were given six mild electric footshocks (0.3 mA) over 3 min (1 every 30 sec) in a mouse shock chamber.

### Adult behavioral tests

All tests were performed in the same sequence (elevated plus maze [EPM], open field, emergence test), at the same age (mean age 99 days) and in the light phase for all mice.

### Elevated plus maze

On day one, animals were tested in the EPM. The EPM was raised 100 cm above the floor, made of black plastic, and comprised two open and opposite arms (28 × 6 cm) and two closed and opposite arms (28 × 6 cm with 14 cm high walls) arranged in a cross shape. The arms were connected by a central square (6 × 6 cm). During testing, an animal was placed in the central square of the maze facing an open arm. Behavior was recorded for 5 min via a video recorder mounted above the maze, and tracking software (Limelight; Actimetrics, Wilmette, IL) was used to analyze the amount of time animals spent in the open versus the closed arms (minus time spent in the central square), and the number of times animals crossed from one arm to another. Data from five male and five female mice which fell off the apparatus before testing was complete were excluded. Numbers used for each experiment can be found in Table 1.

**Table 1:** Number of animals used in each behavioral test

Analysis	Con females	JS females	Con males	JS males
EPM	11	6	7	11
Open field	11	11	11	12
Emergence	11	11	11	12

Con, control; JS, juvenile stress; EPM, elevated plus maze.

### Open field

Twenty-four hours after testing in the EPM, animals were tested in the open field. The open field consisted of a white plastic box (50 × 50 × 15 cm high) divided into 16 equal sized squares. During testing, animals were placed into the center of the open field, and filmed for 5 min via a video camera mounted above the maze. Tracking software (Limelight; Actimetrics) was used to analyze the number of crossings animals made between the 16 squares, and the percentage of time spent in the central four compared to the outer 12 squares of the maze.

### Emergence test

Twenty-four hours after testing in the open field, animals were tested in the emergence test. The apparatus was made of Perspex, and consisted of two compartments, one covered and dark (15 × 17 × 26.5 cm, 0.01 lux), the other light and open (27 × 26.5 × 26.5 cm, 66 lux). A sliding door connected the two. Animals were placed into the dark compartment, given 1 min to settle, the door was raised and time to emerge into the light compartment was recorded. This is another test of anxiety behavior in rodents (Frye et al. 2000; Walf et al. 2009).

### Tissue extraction

One week after behavioral testing, mice were killed by CO_2_ and brains removed and snap frozen for hippocampal mRNA extraction.

### Real time-polymerase chain reaction

The QIAGEN RNeasy system (QIAGEN Ltd., Crawley, U.K.) was used to extract total hippocampal RNA, which was reverse transcribed using Promega reverse transcription kit (Promega UK Ltd., Southampton, U.K.). Triplicate samples of cDNA (the equivalent of 1 ng of total RNA) were incubated with fluorescent probes (using predesigned systems from Applied Biosystems [Warrington, U.K.]) and gene-specific primers (GR [NR3C1]: forward 5′-GTGGAAGGACAGCACAATTACCT-3′ and reverse 5′-GCGGCATGCTGGACAGTT-3′, MR [NR3C2]: forward 5′-CCCTACCATGTCCTAGAAAAGC-3′ and reverse: 5′-AGAACGCTCCAAGGTCTGAG-3′) in 1x Roche LightCycler 40 probes mastermix (Roche Diagnostics, West Sussex, U.K.). A Roche LightCycler 480 was used for polymerase chain reaction (PCR) cycling and detection of fluorescent signal, and a serial dilution of cDNA pooled from all samples was used to create a standard curve for each primer–probe set. Results were standardized using the housekeeping gene HPRT1 (forward sequence: 5′-TCCTCCTCAGACCGCTTTT-3′, reverse sequence: 5′-CCTGGTTCATCATCGCTAATC-3′).

### Data analysis

Data were analyzed by linear models. All data were checked for normality of distribution and homogeneity of variance and were transformed to provide the best approximation to a normal distribution when in violation of these assumptions (Box and Cox 1964). The first two models investigated the effects of group (Con, JS), sex, and group × sex interaction on the percentage of time spent in the open arms and number of crossing made in the EPM. A third and fourth model looked at the effects of group, sex, and group × sex interaction on percentage of time spent in the center and number of crossing made in the open field maze. Another model investigated the effect of group, sex, and group × sex on time to emerge from the dark to the light side of the emergence box. Separate models were set up to investigate the effects of group, sex, and group × sex on the hippocampal mRNA expression levels of HPRT1, GR, MR, and GR:MR ratio. Between one and five animals were used per litter, so litter was nested within group, and fitted as a random factor in all models to account for litter effects.

## Results

**Figure 1:**
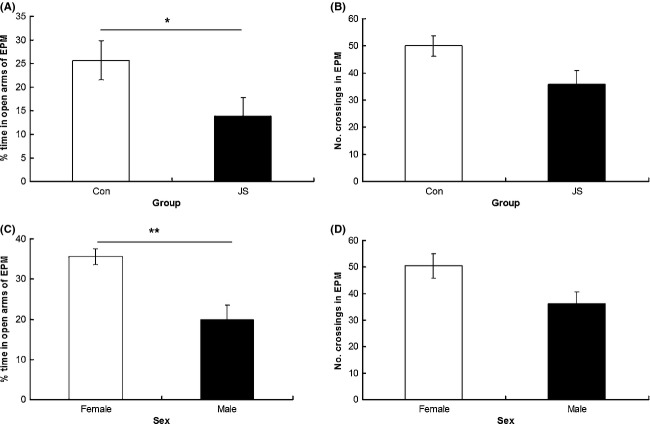
Elevated plus maze. (A) Percentage of time spent in the open arms and (B) number of crossings made in the elevated plus maze (EPM) by control (Con) and juvenile stress (JS) animals. (C) Percentage of time spent in the open arms and D) number of crossing made in the EPM by female and male animals. Error bars represent 1 SE, bars connected by an asterisk are significantly different to one another. (*P < 0.05, **P < 0.01, ***P < 0.001).

**Figure 2:**
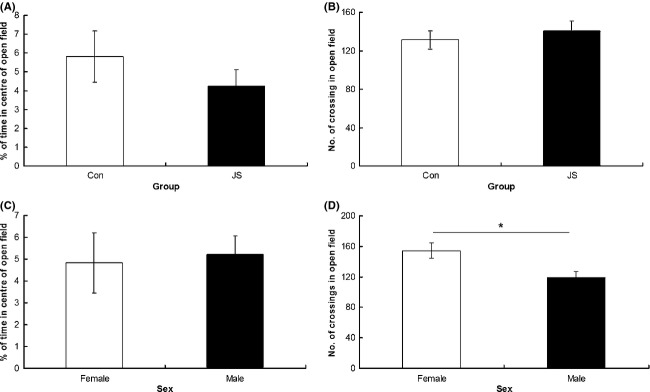
Open field test. (A) Percentage of time spent in the center of the open field maze and (B) number of crossings made in the open field by control (Con) and juvenile stress (JS) animals. (C) Percentage of time spent in the center of the open field maze and (D) number of crossings made in the open field by female and male animals. Error bars represent 1 SE, bars connected by an asterisk are significantly different to one another. (*P < 0.05, **P < 0.01, ***P < 0.001).

**Figure 3:**
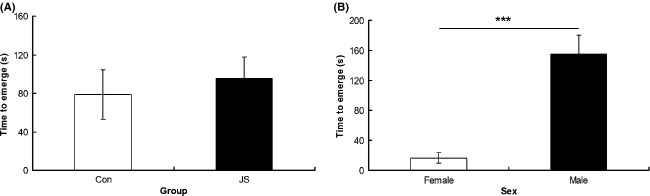
Emergence test. Time to emerge from the emergence box for (A) control (Con) and juvenile stress (JS) and (B) female and male animals. Error bars represent 1 SE, bars connected by an asterisk are significantly different to one another. (*P < 0.05, **P < 0.01, ***P < 0.001).

As a group, JS animals spent a greater proportion of time in the closed arms of the EPM than control animals (*F*_1,8.28_ = 9.17, *P* = 0.02, data square root transformed, Fig. 1A), and there was no interaction between group × sex on percentage of time in the closed arms (*F*_1,28.13_ = 3.67, *P* = 0.7). There was no difference between groups (*F*_1,9.19_ = 1.86, *P* = 0.21), and no group × sex interaction (*F*_1,29.88_ = 0.29, *P* = 0.6) on number of arm entries made in the EPM task (Fig. 1B). There was no difference between groups (*F*_1,17.68_ = 0.87, *P* = 0.36) or a group × sex interaction (*F*_1,39.75_ = 1.92, *P* = 0.17) on percentage of time spent in the center of the open field (Fig. 2A), and no difference between groups (*F*_1,12.5_ = 0.8, *P* = 0.39) or a group × sex interaction (*F*_1,36.83_ = 0.43, *P* = 0.52) on number of crossings in the open field (Fig. 2B). Similarly, there was no difference between groups (*F*_1,11.5_ = 0.55, *P* = 0.47) or a group × sex interaction (*F*_1,35.42_ = 0.13, *P* = 0.72) on time to emerge from the emergence box (Fig. 3A).

Females showed less anxiety-related behavior than males, spending a greater proportion of time on the open arms of the EPM (*F*_1,28.13_ = 8.67, *P* < 0.01, data square root transformed, Fig. 1C), and showing a trend toward making more arm entries in the EPM (*F*_1,29.88_ = 2.88, *P* = 0.09, Fig. 1D), as well as making more crossings in the open field (*F*_1,36.83_ = 6.81, *P* = 0.01, Fig. 2D) and emerging sooner from the dark side of the emergence apparatus (*F*_1,35.42_ = 27.25, *P* < 0.0001 Fig. 3B). However, there was no difference between the sexes in the amount of time spent in the center of the open field (*F*_1,39.75_ = 0.3, *P* = 0.86, Fig. 1C).

**Figure 4:**
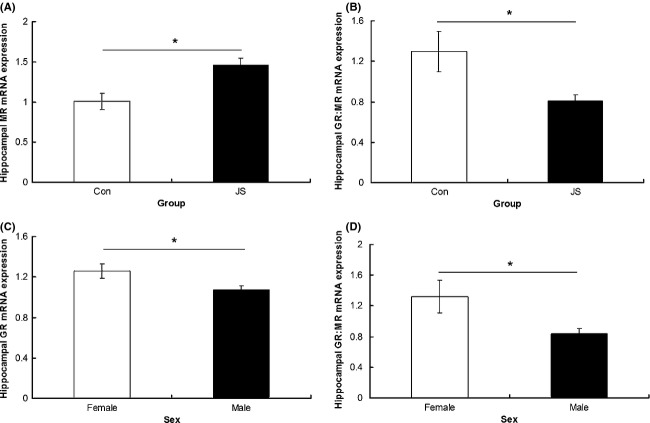
Hippocampal mRNA expression. (A) MR and (B) GR:MR ratio in control (Con) and juvenile Stress (JS) animals. Hippocampal mRNA expression of (C) GR, (D) GR:MR ratio in males and females. Error bars represent 1 SE, bars connected by an asterisk are significantly different to one another. (*P < 0.05, **P < 0.01, ***P < 0.001).

Overall, JS animals had higher expression of hippocampal MR mRNA than controls (*F*_1,2.81_ = 25.13, *P* = 0.02, Fig. 4A) and lower GR:MR ratio (*F*_1,2.58_ = 22.78, *P* = 0.02, data box-cox transformed, Fig. 4B) than control animals. There was no interaction between group and sex on hippocampal MR expression (*F*_1,16.3_ = 0.01, *P* = 0.91) or GR:MR ratio (data box-cox transformed, *F*_1,16.15_ = 0.003, *P* = 0.95). There was no difference between control and JS animals in hippocampal GR expression (*F*_1,9.48_ = 0.23, *P* = 0.64) and no group × sex interaction (*F*_1,19.94_ = 0.26, *P* = 0.62).

Females expressed greater hippocampal mRNA levels of GR (*F*_1,19.94_ = 5.88, *P* = 0.02, Fig. 4C) and a higher GR:MR ratio (*F*_1,16.15_ = 7.04, *P* = 0.02, data box-cox transformed, Fig. 4D) than males. There were no sex differences in hippocampal MR expression (*F*_1,16.3_ = 2.9, *P* = 0.1).

There were no differences in the expression of the housekeeping gene, HPRT1, between groups (*F*_1,8.59_ = 1.33, *P* = 0.28), sexes (*F*_1,14.38_ = 0.17, *P* = 0.69), or group × sex interaction (*F*_1,14.38_ = 0.48, *P* = 0.5).

## Discussion

### Juvenile stress

The experience of stress in the juvenile phase increased anxiety-like behavior on the EPM in adulthood. This result is in agreement with previous studies in rats, (Avital and Richter-Levin 2005; Tsoory et al. 2007; Ilin and Richter-Levin 2009), suggesting that the effects of JS on anxiety are conserved across rats and mice. However, it contrasts with results from one study using mice, in which no effects of JS were found on adulthood behavior in an EPM (Peleg-Raibstein and Feldon 2011). One possible explanation for the disagreement between studies is differences in the type and duration of JS protocols used, as well as sample sizes. In particular, the previous study used 5 days of variable stress (including exposure to a shaking platform, water deprivation, and exposure to a predator, stressors not used in the present study) whereas 3 days were used in this study. Furthermore, sample sizes were significantly smaller (*n* = 7/group) in the Peleg-Raibstein and Feldon (2011) study, whereas the present study used 18 control and 17 stressed animals (combined over males and females). Our result also reflects the findings in human populations, in which juvenile or childhood stress shows a strong correlation with anxiety disorders in adulthood (Green et al. 2010). The specific mechanisms underlying these disruptions are not well understood, although existing studies suggest that reprogramming of the HPA axis may be involved (Meaney et al. 2007; McGowan et al. 2009; Belay et al. 2011; van Hasselt et al. 2012). Therefore, we investigated hippocampal mRNA expression of genes involved in the stress response (specifically, CRs) in adult animals that had experienced JS.

Compared to control animals, hippocampal MR mRNA expression was upregulated in adults that had experienced JS, and the GR:MR ratio was lower. Previous studies have revealed mixed results regarding the effects of stress on corticosteroid expression in the hippocampus (Welberg et al. 2001). Acute forced swim and novelty exposure increased MR expression in the hippocampus 24 h later in adult rats (Reul et al. 2000), and neonatal stress increased hippocampal MR expression and anxiety behavior in adulthood (Gill et al. 2012). In contrast, predator stress in adulthood decreased hippocampal MR expression 4 months later (Wang et al. 2012), and environmental enrichment restored chronic cerebral hypoperfusion induced reductions in hippocampal MR and GR in adult rats (Zhang et al. 2013). Furthermore, exposure to stress in the prenatal period resulted in decreased MR and GR expression in the hippocampus, and increased GR expression in the amygdala in adulthood (Levitt et al. 1996). The discrepancies between studies are likely due to differences in experimental protocols as well as timing and type of stress exposure.

Glucocorticoid receptors and MR are involved in regulating the stress response via the HPA axis, and are abundantly expressed in the hippocampus (Reul et al. 2000). Nuclear MR has a high affinity for glucocorticoids, and is thought to maintain the stress response, setting thresholds for its activation (vanHaarst et al. 1997; Joels et al. 2008). Membrane bound MR has a lower affinity for glucocorticoids, and is thought to mediate fast nongenomic actions, playing a crucial role at the onset of the stress reaction (Karst et al. 2005; Joels et al. 2008). Specifically, in the hippocampus, nongenomic presynaptic MR increases excitability through promoting glutamate release, and postsynaptic nuclear MR enhances potential probability (Karst et al. 2005; Joels et al. 2008). Following this, GR-mediated mechanisms dampen the initial stress response, normalizing brain activity and promoting recovery, with nonnuclear postsynaptic GR receptors decreasing excitation (Joels et al. 2008). In the present experiment, increased levels of MR in the hippocampus of stressed animals could result in a greater magnitude of initial stress response, with the lower GR:MR ratio resulting in a decreased magnitude of or longer duration to GR-mediated dampening. This could be a potential mechanism underlying the increased anxiety behavior observed in this model, although further experiments are needed to investigate this hypothesis further. In agreement with these findings, blocking the action of MR receptors with an antagonist has been found to decrease anxiety behavior in rats (Smythe et al. 1997), and MR/GR imbalances have been found in patients with psychiatric disorders (Baes et al. 2012). Some studies have found increased GR and MR expression in depressed individuals, others decreased, and it has therefore been suggested that any alteration in these receptors should be considered as a biomarker of disease (McGowan et al. 2009; Berardelli et al. 2013; Medina et al. 2013). Furthermore, human carriers of certain MR alleles are more reactive to stress, showing enhanced amygdala activation and HPA activation in response to stress (van Leeuwen et al. 2011; Bogdan et al. 2012).

It should be noted that behavioral alterations between JS and control animals were only found in one measure of anxiety behavior, the EPM, and not in two subsequent tests (open field and emergence test). A possible reason for this is that experience of the EPM (first test encountered) could have affected subsequent performance in the open field and emergence tests, and suggests caution when performing multiple behavioral tests on the same animal, something which remains an unresolved issue in behavioral test batteries (Paylor et al. 2006; Blokland et al. 2012). Alternatively, it has been suggested that these three tests measure subtly different aspects of anxiety-like behavior (Ramos 2008), with the current results suggesting a selective deficit on the EPM. A further option is that stress effects on anxiety are subtle, with effects seen in only one out of the three tests performed.

### Sex differences

Sex differences were found in all three behavioral tests performed, with female animals displaying lower levels of anxiety-like behavior and greater levels of activity. Female mice and rats typically display less anxiety than males in the EPM (Zimmerberg and Farley 1993; Voikar et al. 2001). In the present study, we find that hippocampal GR expression is higher in females, suggesting a role for CRs in differences in anxiety behavior between the sexes. This result adds to recent findings of sex differences in forebrain GR (including hippocampus) on HPA axis regulation and depression-type behaviors (Solomon et al. 2012). As sex differences are found in the development of neuropsychiatric disorders (Bao and Swaab 2010), this highlights further that males and females need to be considered separately in basic research models, and suggests different MR/GR between sexes may contribute to sex differences in vulnerability to stress-related disorders.

## Conclusion

Experiencing stress in the prepubertal or juvenile phase increased anxiety-like behavior and altered the expression of MR and GR:MR in the hippocampus in adulthood. This alteration in CR expression provides a potential mechanism for the observed increase in anxiety-like behavior observed in adulthood. Further evidence for the involvement of CR receptors in adult anxiety-like behavior is provided by the finding that females demonstrated greater GR and GR:MR expression in the hippocampus, with corresponding decreases in anxiety-type behaviors when compared to males. These results demonstrate the potential role of CR in mediating later anxiety-type behavior when stress is experienced early in life.
